# Vitamin B6 in Plasma and Cerebrospinal Fluid of Children

**DOI:** 10.1371/journal.pone.0120972

**Published:** 2015-03-11

**Authors:** Monique Albersen, Marjolein Bosma, Judith J. M. Jans, Floris C. Hofstede, Peter M. van Hasselt, Monique G. M. de Sain-van der Velden, Gepke Visser, Nanda M. Verhoeven-Duif

**Affiliations:** 1 Department of Medical Genetics, University Medical Center (UMC) Utrecht, Utrecht, The Netherlands; 2 Department of Paediatric Metabolic Diseases, Wilhelmina Children’s Hospital, University Medical Center (UMC) Utrecht, Utrecht, The Netherlands; IIBB/CSIC/IDIBAPS, SPAIN

## Abstract

**Background:**

Over the past years, the essential role of vitamin B6 in brain development and functioning has been recognized and genetic metabolic disorders resulting in functional vitamin B6 deficiency have been identified. However, data on B6 vitamers in children are scarce.

**Materials and Methods:**

B6 vitamer concentrations in simultaneously sampled plasma and cerebrospinal fluid (CSF) of 70 children with intellectual disability were determined by ultra performance liquid chromatography-tandem mass spectrometry. For ethical reasons, CSF samples could not be obtained from healthy children. The influence of sex, age, epilepsy and treatment with anti-epileptic drugs, were investigated.

**Results:**

The B6 vitamer composition of plasma (pyridoxal phosphate (PLP) > pyridoxic acid > pyridoxal (PL)) differed from that of CSF (PL > PLP > pyridoxic acid > pyridoxamine). Strong correlations were found for B6 vitamers in and between plasma and CSF. Treatment with anti-epileptic drugs resulted in decreased concentrations of PL and PLP in CSF.

**Conclusion:**

We provide concentrations of all B6 vitamers in plasma and CSF of children with intellectual disability (±epilepsy), which can be used in the investigation of known and novel disorders associated with vitamin B6 metabolism as well as in monitoring of the biochemical effects of treatment with vitamin B6.

## Introduction

**Fig 1:**
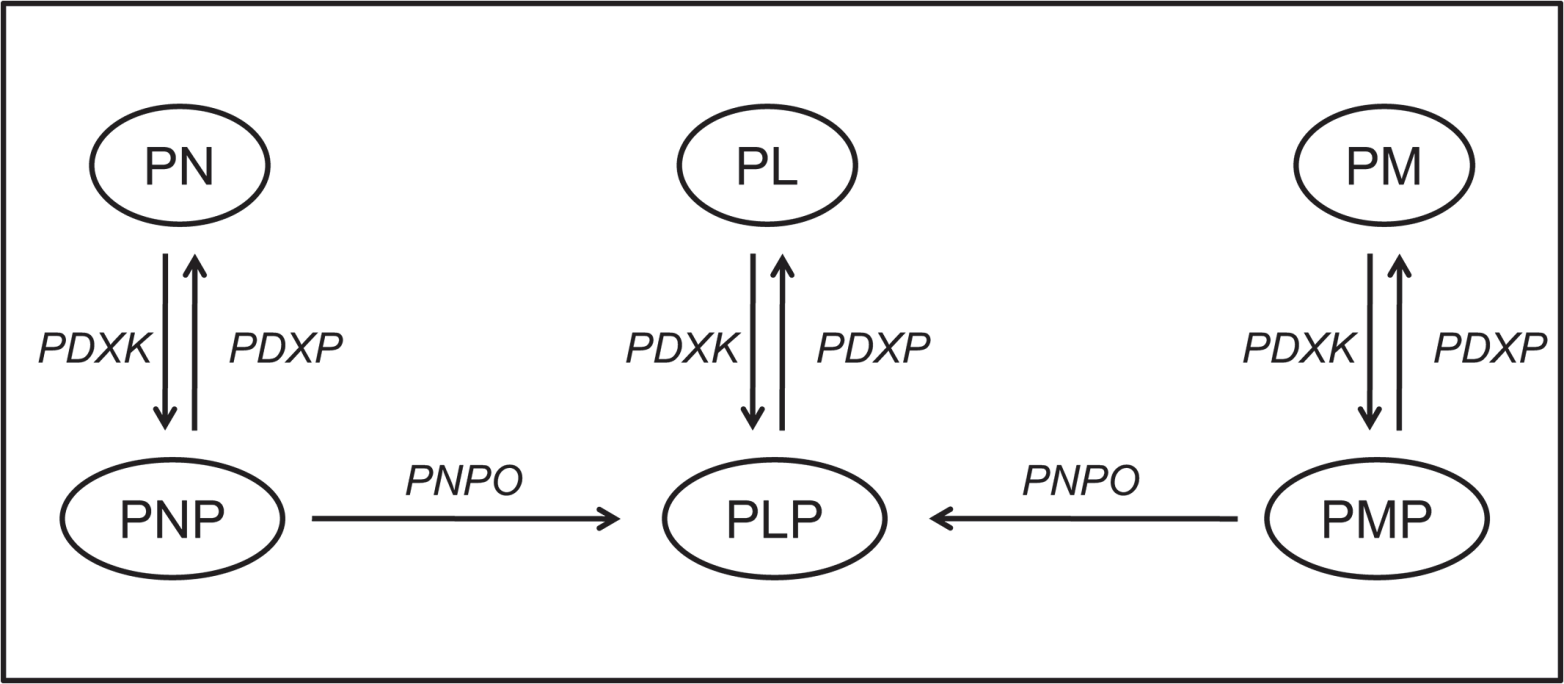
Human vitamin B6 metabolism. PDXK = pyridoxal kinase. PDXP = vitamin B6-specific phosphatase. PNPO = pyridox(am)ine phosphate oxidase.

Vitamin B6 consists of six different vitamers: the alcohol pyridoxine (PN), the aldehyde pyridoxal (PL), the amine pyridoxamine (PM) and their phosphate-esterified forms (**Fig. 1**). Pyridoxal phosphate (PLP) is the active B6 vitamer, which is produced from its precursor vitamers by phosphorylation (of PN, PM and PL) and oxidation (of PN phosphate and PM phosphate), through the actions of pyridoxal kinase and pyridox(am)ine phosphate oxidase (PNPO), respectively. For transport across the cell membrane, a vitamin B6-specific phosphatase enzyme is indispensable (Fig. 1). Vitamin B6 is broken down to pyridoxic acid (PA), which is excreted in urine. PLP is well known for its cofactor function in numerous enzymatic reactions in the central nervous system, where it is highly important in dopamine, serotonin, glutamate, γ-aminobutyrate, glycine and D-serine metabolism.

Over the past years, the essential role of vitamin B6 in normal brain development and functioning has been recognized. Specific genetic metabolic disorders resulting in a functional deficiency of vitamin B6 have been identified as an underlying cause of vitamin B6-responsive epilepsy in children. Most patients suffering from PNPO (OMIM #610090) [1] and antiquitin (α-AASA dehydrogenase; OMIM #266100) [2] deficiencies are severely neurologically affected. The epilepsy resulting from these disorders can be treated with vitamin B6. Additional causes of a functional deficiency of vitamin B6 are hypophosphatasia (alkaline phosphatase (ALPL) deficiency; OMIM #241500) [3,4] and hyperprolinaemia type II (pyrroline-5-carboxylate dehydrogenase deficiency; OMIM #239510) [5]. Diagnosis can be difficult, because biochemical abnormalities are not always present [1,6–8] and the response to treatment can be nondirective [9,10].

Optimal treatment strategies for PNPO and antiquitin deficiencies are not known. Whereas antiquitin deficiency can be treated with both PN and PLP, PNPO deficiency usually needs PLP treatment, although patients responsive to PN have been recently reported [9,10]. A substantial number of affected children have intellectual disability, despite early treatment with high doses of PN or PLP [11,12]. In healthy individuals, ingestion of large doses of PN has been reported to be toxic [13,14] and to result in polyneuropathy [14]. The mechanism of this neurotoxicity has not been elucidated.

In literature, data on B6 vitamers in children are scarce [15–22]. Recently, Footitt et al [18] presented concentrations of all B6 vitamers in plasma of children. In addition, decreased concentrations of PLP [1,17,23–25] and/or PL [1] in cerebrospinal fluid (CSF) of a few patients suffering from PNPO or antiquitin deficiency have been published. Neither concentrations of the other B6 vitamers in CSF nor concentrations of any of the B6 vitamers in plasma of these patients were reported, while these might be abnormal as well.

In this study, we determined concentrations of all B6 vitamers in plasma and CSF of children and we investigated the influence of sex and age. Given the fact that analysis of B6 vitamers is of particular importance in patients suffering from epilepsy, we also studied the influence of epilepsy and treatment with anti-epileptic drugs (AEDs). Our approach to simultaneously sample plasma and CSF allows presentation of the relationship between the vitamers in both body fluids.

## Materials and Methods

For this cross-sectional study, we used remnant plasma and CSF samples that were taken for diagnostic purposes. Plasma and/or CSF were sampled from 70 children (1–18 years of age) visiting the Sylvia Tóth Center (Wilhelmina Children’s Hospital, University Medical Center Utrecht, The Netherlands) for diagnostic evaluation of intellectual disability (**S1 Table**). Plasma was obtained by venous sampling (4 mL in heparin tube) and subsequent centrifugation (3000 rpm, 5 min). For most children, CSF was also obtained. This was done on the same day, by lumbar puncture (fraction IV) [22]. An additional set of remnant CSF samples obtained from 35 epileptic children using one or more AEDs (1–18 years of age; Sylvia Tóth Center and other samples) were used to study the influence of AED therapy on B6 vitamer concentrations in CSF (S1 Table).

After withdrawal, samples had been protected from light and stored at −80°C until analysis. The longest time to analysis was 6 years and 3 months; B6 vitamers are known to be stable during storage at −80°C [26,27] for at least 15 months [26]. B6 vitamer (PN, PL, PLP, PM, PMP) and PA concentrations in plasma and CSF were determined by ultra performance liquid chromatography-tandem mass spectrometry (UPLC-MS/MS) [22,28]. This method was developed and validated in our laboratory and has been described in detail by van der Ham et al [22] (for CSF) and Albersen et al [28] (for plasma). In short, we use a Xevo-TQ MS triple quadropole mass spectrometer with an electrospray ionization source and an Acquity UPLC (Waters, Manchester, UK). B6 vitamers and PA are separated on an Acquity HSS-T3 UPLC column (Waters, Massachusetts, USA) using a buffer containing acetic acid, heptafluorobutyric acid and acetonitrile. Our method requires a simple sample preparation procedure of protein precipitation using trichloroacetic acid containing isotope-labeled internal standards. Positive electrospray ionization is used to monitor transitions. Total measurement time per sample is only 3.5 minutes and inter-assay variations range from 4.5 to 25% [22,28].

Statistical analyses were performed using SPSS 20.0 (IBM Corporation). Because none of the B6 vitamers in plasma and CSF, nor their unstandardized residuals, showed a normal distribution, nonparametric tests (Mann-Whitney U (comparison of two groups) and Mann-Whitney U with Bonferroni correction (comparison of more than two groups)) were applied to study differences in B6 vitamer concentrations. Spearman’s rho (ρ) was used to describe correlations.

### Ethics Statement

Written parental informed consent was obtained for the use of all remnant samples. Approval by the Medical Ethics Committee of the UMC Utrecht was obtained.

## Results

Concentrations of PL, PLP, PM and PA were determined in both plasma and CSF. Since PMP in plasma is highly instable, quantification was not possible [28]. PMP in CSF, PM in plasma and PN in plasma and CSF were below the limit of quantification (LOQ; 5.4 nmol/L for PMP in CSF, 2.7 nmol/L for PM in plasma and 0.3 and 0.03 nmol/L for PN in plasma and CSF, respectively) [22,28].

Three children with extreme B6 vitamer concentration(s) in plasma and/or CSF were excluded (S1 Table). In one of these children, PN was present in CSF (0.7 nmol/L), as a consequence of vitamin B6 supplementation [22]. In the other two children, concentrations of one or more B6 vitamers were >1.5 times lower or higher than the lower or upper limit of the observed range. In one child, we found decreased CSF PLP, and the other child showed elevated PA in plasma and CSF. Extensive metabolic investigations did not yield a diagnosis and the results of genetic analysis in these two children are pending.

Therefore, concentrations of PM (in CSF), PL, PLP and PA (in plasma and CSF) of 67 children were studied in more detail. In two children, plasma and CSF were not withdrawn on the same day, but 21 and 22 days apart. Relative B6 vitamer concentrations did not differ from those in the other 65 children.

### B6 vitamer concentrations in plasma and CSF

**Table 1** displays median B6 vitamer concentrations in plasma and CSF of children. The most abundant vitamer in plasma was PLP (median concentration 33.9 (range 20.5–151) nmol/L), whereas in CSF the concentration of PL (28.2 (16.1–55.7) nmol/L) was the highest.

**Table 1:** Concentrations of PM, PL, PLP and PA (nmol/L) and their ratios and correlations in plasma (n = 42) and/or CSF (n = 41) of children (1–18 years).

*B6 vitamer concentration (nmol/L)*	Body fluid		Median	Range	
**PM**	***CSF***	0.5	0.3–0.9	
**PL**	***Plasma***	21.1	8.8–58.7	
	***CSF***	28.2	16.1–55.7	
		*Boys*	24.3	16.1–55.7	
		*Girls*	33.1	21.1–45.9	
**PLP**	***Plasma***	33.9	20.5–151	
	***CSF***	18.2	11.0–33.7	
**PA**	***Plasma***	24.7	8.8–104	
	***CSF***	0.9	≤0.09^a^–3.1	
***B6 vitamer ratio***	**Body fluid**	**Median**	**Range**	**Correlation (rho (ρ))** ^**b**^
**PL:PM**	***CSF***	70.3	27.7–154	0.090
**PLP:PL**	***Plasma***	1.9	1.0–4.2	0.622**
	***CSF***	0.6	0.4–1.4	0.539**
**PLP:PM**	***CSF***	40.3	16.5–108	−0.042
**PA:PL** ^**c**^	***Plasma***	1.3	0.4–3.7	0.514**
	***CSF***	0.03	0.00–0.07	0.565**
**PA:PLP** ^**c**^	***Plasma***	0.7	0.2–2.6	0.614**
	***CSF***	0.05	0.01–0.14	0.279
**PA:PM** ^**c**^	***CSF***	2.0	0.2–7.3	−0.103

^a^ LOQ of this B6 vitamer [22].

^b^ Spearman’s rho (ρ).

^c^ For ratio calculations, PA concentrations <LOQ were replaced by the determined LOQ of PA (*n* = 2).

** = significant at the p<0.001 level.

PM = pyridoxamine. PL = pyridoxal. PLP = pyridoxal phosphate. PA = pyridoxic acid. CSF = cerebrospinal fluid.

### B6 vitamer ratios and correlations in and between plasma and CSF

**Fig 2:**
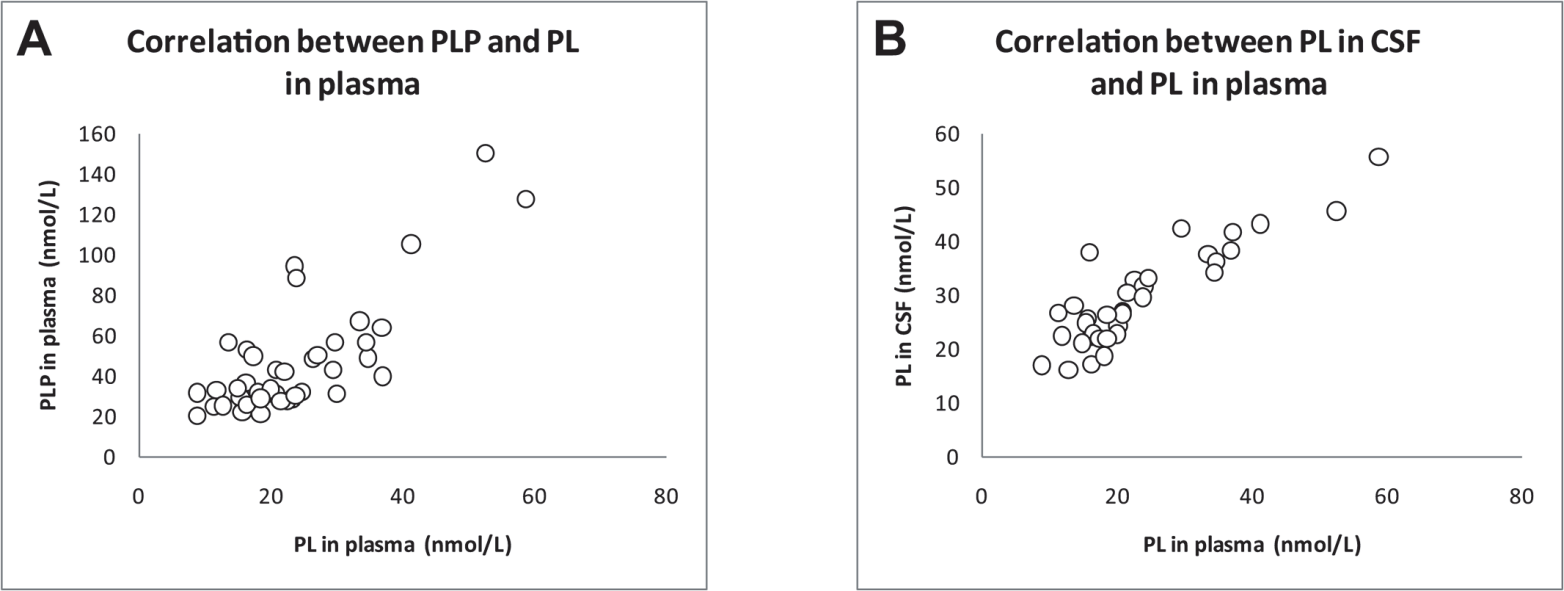
A. Correlation (Spearman’s rho, p-value) between PLP and PL in plasma (ρ = 0.622, p<0.001) of children (n = 42). **B. Correlation (Spearman’s rho, p-value) between PL in CSF and PL in plasma (ρ = 0.806, p<0.001) of children (n = 35)**.

Table 1 shows ratios as well as correlations between PM, PL, PLP and PA in plasma and/or CSF of children. In plasma, the strongest correlation was observed between PLP and PL (ρ = 0.622, p<0.001; **Fig. 2A**). In CSF, concentrations of PA and PL were most strongly correlated (ρ = 0.565, p<0.001).

In **Table 2**, ratios and correlations are shown for PL, PLP and PA between CSF and plasma. The strongest correlation was found for PL (ρ = 0.806, p<0.001; **Fig. 2B**).

**Table 2:** Ratios and correlations of PL, PLP and PA between CSF and plasma of children (1–18 years; n = 35).

*B6 vitamer CSF*:*plasma*	Median	Range	Correlation (rho (ρ)^b^)
**PL**	1.3	0.9–2.4	0.806**
**PLP**	0.5	0.2–0.8	0.524*
**PA** ^**a**^	0.03	0.00–0.28	0.226

^a^ For ratio calculations, PA concentrations <LOQ were replaced by the determined LOQ of PA (*n* = 2)

^b^ Spearman’s rho (ρ).

* = significant at the p<0.005 level.

** = significant at the p<0.001 level.

CSF = cerebrospinal fluid. PL = pyridoxal. PLP = pyridoxal phosphate. PA = pyridoxic acid.

### Influence of sex and age

Median concentrations of PL in CSF were lower in boys than in girls (24.3 (16.1–55.7) versus 33.1 (21.1–45.9) nmol/L, p = 0.014) (Table 1). Concentrations of the other B6 vitamers in plasma and CSF did not differ between sexes (data not shown).

Concentrations of PL and PLP in CSF marginally decreased from one to 18 years of age (ρ = −0.379, p = 0.015 for PL; ρ = −0.338, p = 0.031 for PLP). B6 vitamer concentrations in plasma did not correlate with age (data not shown).

### Effect of epilepsy and treatment with anti-epileptic drugs (AEDs)

Of all 67 children, 37 did not have epilepsy nor used any AEDs, 11 did have epilepsy for which they did not use any AEDs and 19 had epilepsy and used one or more AEDs (S1 Table). B6 vitamer concentrations in plasma and concentrations of PM, PL and PA in CSF did not differ between these groups. Median PLP concentrations, however, were lower in CSF of children using one or more AEDs (14.8 (11.5–27.4) nmol/L) than in CSF of children with untreated epilepsy (19.4 (13.5–33.7) nmol/L, p = 0.016).

To validate this finding, B6 vitamer concentrations in an additional set of CSF samples obtained from children using one or more AEDs (1–18 years of age; *n* = 35) were compared with B6 vitamer concentrations in CSF of the original set of (non-)epileptic children not using any AEDs, since in our dataset there was no influence of epilepsy itself (total CSF sample *n* = 41) (S1 Table). The original set of children with AED therapy was not included in this analysis.

**Fig 3:**
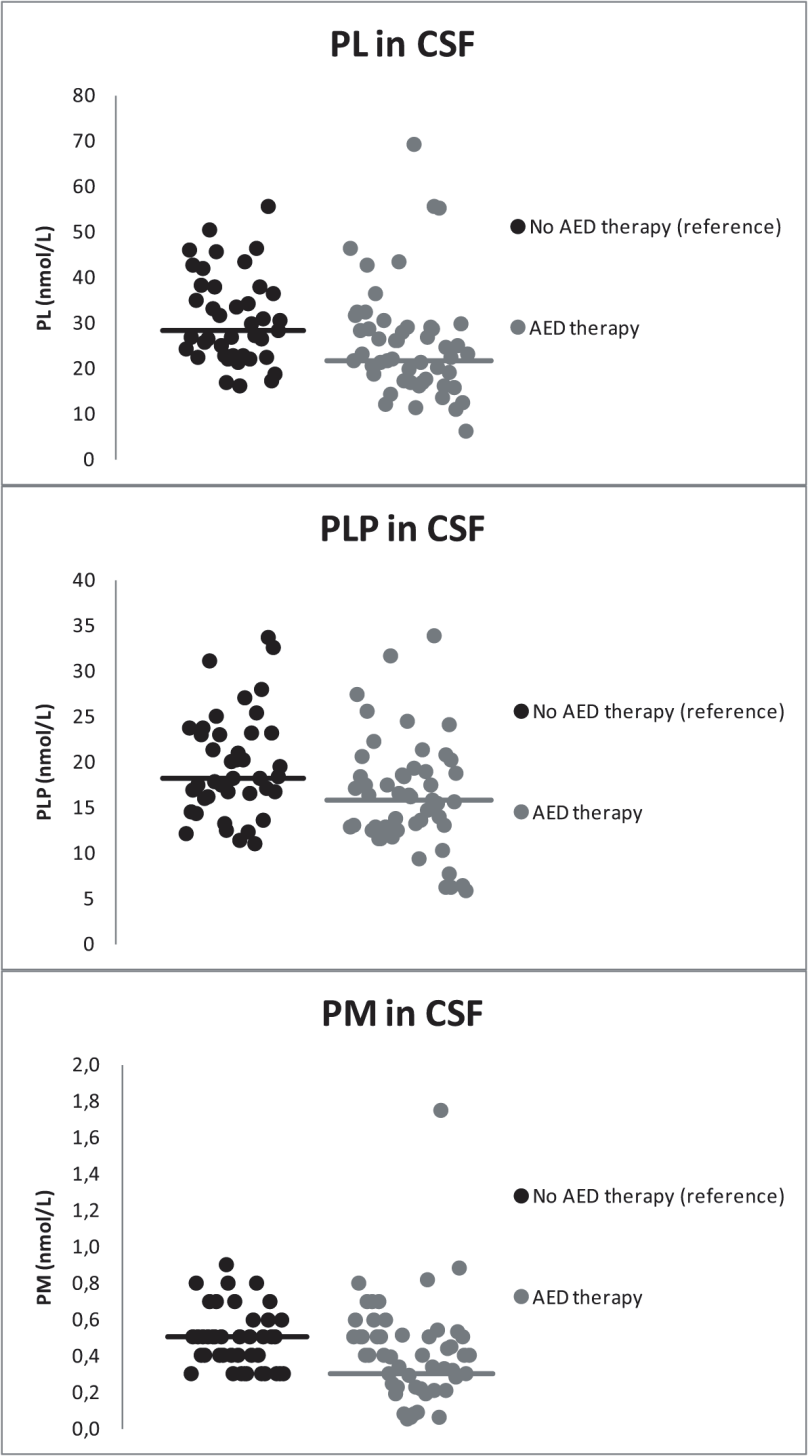
Concentrations of PL, PLP and PM in CSF of children using one or more AEDs (n = 51; from both the original and additional sets) compared to children not using any AEDs (n = 41). Median concentrations of PL, PLP and PM were lower in CSF of children with AED-treated epilepsy than in CSF of children not using any AEDs.

Children with AED-treated epilepsy indeed showed lower median concentrations of PLP in CSF (**Table 3**; **Fig. 3**). In addition, median CSF concentrations of PL as well as PM were lower compared to children not using any AEDs (Table 3; Fig. 3). Median concentrations of PA in CSF did not differ between these groups.

**Table 3:** The effect of treatment with anti-epileptic drugs (AEDs) on B6 vitamer concentrations in CSF of children (1–18 years).

*B6 vitamer in CSF (nmol/L)*	Children with AED-treated epilepsy (*n* = 35)	(Non-)epileptic children not using any AEDs (*n* = 41)	Difference (p-value)
**PM**	0.3 (0.1–1.8)	0.5 (0.3–0.9)	<0.001*
**PL**	21.7 (6.3–69.1)	28.2 (16.1–55.7)	<0.001*
**PLP**	15.7 (5.9–33.8)	18.2 (11.0–33.7)	0.013*
**PA**	1.2 (0.09^a^–5.5)	0.9 (0.09^a^–3.1)	0.098

* = significant at the p<0.05 level.

^a^ LOQ of this B6 vitamer [22].

CSF = cerebrospinal fluid. PM = pyridoxamine. PL = pyridoxal. PLP = pyridoxal phosphate. PA = pyridoxic acid.

In CSF of 9/51 children with AED therapy (from both the original and additional sets), concentrations of PL were below the lower limit of the concentration range observed in children not using any AEDs, whereas PLP in CSF was decreased in seven children. Three of these children showed decreased concentrations of both PL and PLP in CSF. In addition, three children with a decreased concentration of PL in CSF also showed a decreased concentration of PLP in plasma.

Concentrations of PL, PLP and PM in CSF did not differ between types of AED therapy (valproate monotherapy (*n* = 16), valproate combined with at least one other AED (*n* = 14) and monotherapies and/or combinations of other AEDs (*n* = 21); data not shown).

Since AED therapy influenced concentrations of PL, PLP and PM in CSF, and three children displayed decreased concentrations of PLP in plasma, the group of children using one or more AEDs (*n* = 19) was excluded from the original dataset (remaining children *n* = 48; plasma *n* = 42, CSF *n* = 41 and both *n* = 35; S1 Table).

## Discussion

We provide concentrations of all B6 vitamers in simultaneously sampled plasma and CSF of children. Given the ethical concerns, we were unable to obtain CSF samples from healthy children. Therefore, we used remnant CSF samples that were taken for diagnostic evaluation of intellectual disability, which was in some children accompanied by epilepsy. CSF samples taken for other indications like meningitis or encephalitis are not protected from light or stored at −80°C. Thus, our approach to use remnant samples that were taken and stored under strictly prescribed conditions may be the best option. Children visiting the Sylvia Tóth Center, which is a quaternary diagnostic referral institution for children with intellectual disability, undergo extensive metabolic screening. For most children, this includes determination of amino acids, pipecolic acid, homovanillic acid (HVA), 5-hydroxyindoleacetic acid (5-HIAA), 3-O-methyl-DOPA and 5-methyltetrahydrofolate (5-MTHF) in CSF. It therefore is very likely that, when being the cause of the clinical picture in these children, genetic metabolic disorders associated with a functional deficiency of vitamin B6 are picked up by these investigations. To our knowledge, none of the children in our dataset suffered from one of these defects, although we cannot exclude the possibility of an undiagnosed neurometabolic disorder subtly influencing B6 vitamer concentrations. So although we used plasma and CSF of children with intellectual disability, we are confident that our approach provides the best possible reflection of healthy vitamin B6 homeostasis in children and that it enables us to obtain further insight in B6 vitamers and their interrelationships.

In children, like in adults [28], the B6 vitamer composition differs between plasma (PLP>PA>PL) and CSF (PL>PLP>PA>PM). The strong correlations between certain B6 vitamers in plasma and CSF and the strong correlation for especially PL between CSF and plasma, suggest that B6 vitamer concentrations are tightly regulated. Disturbances of B6 vitamer ratios in or between plasma and CSF may therefore indicate possible deficiencies of the enzymes involved in vitamin B6 metabolism or may point towards a problem in vitamin B6 transport from blood to brain [28]. Given that most children with PNPO and antiquitin deficiencies present with seizures during the neonatal and infantile period, it would be of additional value to have knowledge on B6 vitamer concentrations in plasma and CSF of newborns. For CSF, we previously published these values [15]. Plasma samples are not routinely stored at −80°C, unless specifically requested, and are therefore in general not useful for the investigation of B6 vitamers. Regarding our data on B6 vitamer concentrations in CSF of children <1 year of age, postmenstrual age should always be taken into account, since B6 vitamer concentrations in CSF differ between preterm and term newborns and decrease with postmenstrual age [15]. Concentrations in this age group should therefore never be combined with concentrations in children aged 1–18 years.

Interestingly, the correlation between PLP and PL in plasma is different for children (this study) than it is for adults [28]. Plasma concentrations of PL were relatively higher in children, whereas plasma concentrations of PLP were relatively higher in adults. Although both groups cannot be strictly compared to each other, because adults were healthy and fasting [28] and children had intellectual disability, one might hypothesize that age-related changes in the activity of the extracellular ALPL enzyme (OMIM *171760), which hydrolyzes PLP into PL, might underlie this observation, since it is known that plasma ALPL activities are higher in children than in adults [29,30]. The relationship between PLP in plasma and ALPL has been previously shown in a genome-wide association study, in which a single nucleotide polymorphism in the *ALPL* gene (rs1256335) was associated with plasma concentrations of PLP [31].

### Epilepsy and treatment with AEDs

Our study demonstrates that B6 vitamer concentrations in CSF are not influenced by epilepsy. This finding is supported by Footitt et al [17], although they reported CSF concentrations of only PLP. However, since the number of children in our group of epileptic children not using any AEDs is small (*n* = 11), we cannot fully exclude a possible effect of epilepsy on B6 vitamer concentrations in plasma and CSF. We did find lower concentrations of PL and PLP as well as PM in CSF of children with AED therapy. This implicates that AED-treated children are at risk of a deficiency of vitamin B6, which might have adverse effects on their brain development and functioning.

B6 vitamer concentrations in plasma were not influenced by epilepsy nor by AEDs. However, three children with AED therapy showed a decreased concentration of PLP in plasma besides a decreased concentration of PL in CSF. In literature, several AEDs have been associated with decreased concentrations of PLP in plasma, for children [32,33], as well as for adults [34–37].

The group of AED-treated children with low PLP and/or PL concentrations in CSF consisted of children (aged 1.3–16.9 years) with a genetic diagnosis (*n* = 6), which probably does not affect vitamin B6 metabolism, and children without a diagnosis (*n* = 7). In the latter group, extensive metabolic investigations, like mentioned above, did not result in a diagnosis. In none of these children, PNPO and antiquitin deficiency were ruled out by genetic analyses. However, the complex clinical phenotypes were not suggestive of these defects. The children were not treated with anti-tuberculosis medication or antibiotic drugs at the time of plasma and CSF sampling.

### Conclusion

In this study, we provide concentrations of all B6 vitamers in plasma and CSF of children with intellectual disability (±epilepsy). Our data suggest a tight regulation of B6 vitamers in and between plasma and CSF of children. Treatment with anti-epileptic drugs may result in decreased concentrations of several B6 vitamers in CSF. Quantification of vitamin B6 in body fluids is useful in the investigation of known and novel disorders associated with vitamin B6 metabolism and will teach us about the biochemical effects of treatment with vitamin B6.

## Supporting Information

S1 Table Schematic representation of subject numbers in this study. (DOCX)
