# Supplementary material for: Vitamin B6 in Plasma and Cerebrospinal Fluid of Children
Source: PLoS One. 2015 Mar 11;10(3):e0120972. doi: 10.1371/journal.pone.0120972 (PMC4356616; doi:10.1371/journal.pone.0120972)
Supplement: S1 Table — (DOCX) [file pone.0120972.s001.docx]

| Simultaneously sampled plasma and CSF of children (1-18 years of age)  *n*=70 | | | | | |
| --- | --- | --- | --- | --- | --- |
| Exclusion of children with outlier B6 vitamer concentrations in plasma and/or CSF  *n*=3 | | | | | |
| Remaining children  *n*=67 | | | | | |
| No epilepsy  *n*=37 | | Epilepsy without AEDs  *n*=11 | | Epilepsy with AEDs  *n*=19 | |
| Exclusion of children with epilepsy and AEDs  *n*=19 | | | | Plasma  *n*=18 | CSF  *n*=16 (‘original set’) |
| Remaining children  *n*=48 (1.1-17.9 years of age) | | | | Study B6 vitamer concentrations in ‘additional set’ of CSF samples from children with epilepsy and AED treatment  *n*=35  (total *n*=51; 1.1-18.0 years of age) | |
| Plasma  *n*=42 | CSF  *n*=41 | | Both  *n*=35 |  |  |

**S1 Table Schematic representation of subject numbers in this study.**
